# Evogliptin, a dipeptidyl peptidase-4 inhibitor, attenuates pathological retinal angiogenesis by suppressing vascular endothelial growth factor-induced Arf6 activation

**DOI:** 10.1038/s12276-020-00512-8

**Published:** 2020-10-14

**Authors:** Songyi Seo, Mi-Kyung Kim, Ryul-I Kim, Yeongju Yeo, Koung Li Kim, Wonhee Suh

**Affiliations:** 1 Department of Global Innovative Drug, College of Pharmacy, Chung-Ang University, Seoul, 06974 Korea; 2 Drug Discovery Research Laboratories, Dong-A ST Co., Ltd., Gyeonggi-Do, 17073 Korea

**Keywords:** Vascular diseases, Growth factor signalling

## Abstract

Dipeptidyl peptidase-4 (DPP-4) inhibitors are used for the treatment of type 2 diabetes mellitus (DM). Recent studies have shown that beyond their effect in lowing glucose, DPP-4 inhibitors mitigate DM-related microvascular complications, such as diabetic retinopathy. However, the mechanism by which pathological retinal neovascularization, a major clinical manifestation of diabetic retinopathy, is inhibited is unclear. This study sought to examine the effects of evogliptin, a potent DPP-4 inhibitor, on pathological retinal neovascularization in mice and elucidate the mechanism by which evogliptin inhibits angiogenesis mediated by vascular endothelial growth factor (VEGF), a key factor in the vascular pathogenesis of proliferative diabetic retinopathy (PDR). In a murine model of PDR, an intravitreal injection of evogliptin significantly suppressed aberrant retinal neovascularization. In human endothelial cells, evogliptin reduced VEGF-induced angiogenesis. Western blot analysis showed that evogliptin inhibited the phosphorylation of signaling molecules associated with VEGF-induced cell adhesion and migration. Moreover, evogliptin substantially inhibited the VEGF-induced activation of adenosine 5′-diphosphate ribosylation factor 6 (Arf6), a small guanosine 5′-triphosphatase (GTPase) that regulates VEGF receptor 2 signal transduction. Direct activation of Arf6 using a chemical inhibitor of Arf-directed GTPase-activating protein completely abrogated the inhibitory effect of evogliptin on VEGF-induced activation of the angiogenic signaling pathway, which suggests that evogliptin suppresses VEGF-induced angiogenesis by blocking Arf6 activation. Our results provide insights into the molecular mechanism of the direct inhibitory effect of the DPP-4 inhibitor evogliptin on pathological retinal neovascularization. In addition to its glucose-lowering effect, the antiangiogenic effect of evogliptin could also render it beneficial for individuals with PDR.

## Retinal disease: How anti-diabetic drug can help eyes

Pathological retinal angiogenesis, the damaging formation of new blood vessels in the retina, which is associated with various diseases including diabetes, could be reduced using the anti-diabetic drug evogliptin to inhibit the effects of a vascular growth factor. Researchers in South Korea led by Wonhee Suh and Koung Li Kim at Chung-Ang University in Seoul investigated the molecular mechanism underlying evogliptin’s effects. In studies using mice and cultured human cells they found that evogliptin inhibited the activation of signaling molecules that mediate the effects of vascular endothelial growth factor. They also identified an enzyme in the signaling pathway that is directly inhibited by evogliptin. The results offer molecular level insights into the additional benefit gained from using evogliptin to treat diabetes, distinct from the drug’s established effects in lowering blood glucose.

## Introduction

Dipeptidyl peptidase-4 (DPP-4) is a widely expressed enzyme that selectively cleaves peptides with a proline or alanine at the second position after the amino-terminus. DPP-4 substrates, such as glucose-dependent insulinotropic polypeptide and glucagon-like peptide-1 (both incretin hormones), are key regulators of postprandial glucose levels. Hence, gliptins (DPP-4 inhibitors) are used to lower blood glucose levels in type 2 diabetes mellitus (DM)^1^.

Beyond their ability to reduce hyperglycemia, DPP-4 inhibitors have recently shown beneficial effects by mitigating DM-related microvascular complications, such as diabetic retinopathy (DR)^2^. DR is characterized by vascular abnormalities in the retina, wherein increased vascular permeability and reduced blood flow cause retinal ischemia. This retinal ischemia increases the expression of proangiogenic factors, such as vascular endothelial growth factor (VEGF), and results in excessive retinal neovascularization (NV), which is a hallmark of proliferative DR (PDR)^3^. In a preliminary clinical study of patients with type 2 DM, the group treated with DPP-4 inhibitors exhibited a significant decrease in DR progression compared to that of the group treated with sulfonylureas and/or metformin, other antidiabetic drugs, which suggests that DPP-4 inhibitors might have protective effects against DR that are independent of their glucose-lowering effects^4^. Moreover, a cross-sectional study of patients with type 1 DM showed that serum DPP-4 activity in patients with DR was significantly higher than that in patients without DR, which demonstrates the possible association of DPP-4 activity and DR^5^. Another clinical trial study reported that treatment with saxagliptin normalized retinal capillary flow and improved retinal vascular function in patients with type 2 DM^6^. Although these clinical data imply that DPP-4 inhibitors may exert direct beneficial effects against DR independent of their improvement of glycemic control, not much experimental evidence indicating the mechanism by which DPP-4 inhibitors prevent retinal vascular pathogenesis in PDR is available.

Hence, the present study aimed to examine the direct effect of a DPP-4 inhibitor on pathological retinal NV and elucidate the mechanism by which this DPP-4 inhibitor inhibits angiogenesis mediated by VEGF, a key factor in the vascular pathogenesis of PDR. For this purpose, we investigated whether the intravitreal administration of evogliptin, a potent DPP-4 inhibitor that has been clinically used as an oral agent for type 2 DM, could attenuate pathological retinal NV in a murine model showing the vascular pathogenic characteristics of PDR^7,8^. In addition, we examined how evogliptin interferes with the VEGF-induced angiogenic signaling pathway in human primary endothelial cells.

## Materials and methods

### Animals

All experiments were approved by the Institutional Animal Care and Use Committee of Chung-Ang University. Nine- to ten-week-old male C57BL/6J mice and pregnant C57BL/6J mice were purchased from Orient Co., Ltd. (Seoul, Korea) and cared for in accordance with the Guide for the Care and Use of Laboratory Animals published by the United States National Institutes of Health (NIH). The animals were housed in microisolator cages on individually ventilated cage racks lined with aspen shavings (Northeastern Products Corp., Warrensburg, NY), given ad libitum access to an autoclaved standard rodent diet (LabDiet 5008, Purina, St. Louis, MO) and maintained under a 12 h:12 h light/dark cycle. Euthanasia was performed by cervical dislocation under anesthesia. Anesthesia was performed via an intraperitoneal injection of ketamine hydrochloride (100 mg/kg body weight) and xylazine hydrochloride (6 mg/kg body weight). The pupils of the anesthetized mice were dilated using topical drops of 1% tropicamide (Santen, Osaka, Japan).

### Oxygen-induced retinopathy (OIR)

OIR was induced in mice using the protocol reported by Connor et al.^9^. On postnatal day 7 (P7), newborn pups, along with the nursing mothers, were placed in a 75% oxygen chamber connected to an oxygen controller (ProOx P110; BioSpherix, Parish, NY) and remained in the chamber for 5 days. On P12, the pups were then returned to room air with a normal oxygen content and given an intravitreal injection of evogliptin (provided by Dong-A ST Co., Ltd., Korea; 10 µg in 1 µL of dimethyl sulfoxide (DMSO)) or an equivalent volume of DMSO (contralateral control). The mice were sacrificed on P17, and their eyes were enucleated and fixed in 4% (w/v) paraformaldehyde. Underdeveloped neonatal mice with a weight less than 6 g at P17 were excluded. The retinae were then dissected and stained overnight with Alexa Fluor^®^ 594-conjugated *Griffonia simplicifolia* isolectin B4 (IB4; 1:100 dilution; Invitrogen, Carlsbad, CA) at 4 °C. Retinal flat mounts were generated and imaged using a fluorescence microscope (Olympus, Tokyo, Japan); the exposure and gain were kept constant for all samples. In each whole-mount image, the number of pixels in the neovascular tufts was measured using ImageJ software (NIH, Bethesda, MD) and compared to the total number of pixels in the entire retina as reported previously^9^.

### Laser-induced choroidal NV (CNV)

Laser-induced CNV was induced in mice using the protocol reported by Lambert et al.^10^. Briefly, nine- to ten-week-old male C57BL/6J mice were anesthetized, and their pupils were dilated. A Micron IV image-guided laser system (Phoenix Research Laboratories, Pleasanton, CA) was used to create CNV lesions at the 3, 6, 9, and 12 o’clock positions of the posterior pole of the fundus at equal distances from the optic nerve head with the following parameters: wavelength, 532 nm; diameter, 50 μm; duration, 70 ms; and intensity, 220 mW. Only burns for which a bubble was produced at the time of laser treatment were included in the study. Immediately after CNV induction, the mice were given an intravitreal injection of evogliptin (10 µg in 1 µL DMSO) or an equivalent volume of DMSO (contralateral control). Fourteen days after the laser treatment, the mice were anesthetized for further analysis. For the oral administration experiment, evogliptin was suspended in solution containing 0.5% methylcellulose (Sigma-Aldrich, St. Louis, MO), and the mice received an evogliptin suspension (20 mg/kg/day) or an equivalent volume of 0.5% methylcellulose solution by oral gavage (twice daily). Oral gavage was started on the day of CNV development and continued for 2 weeks before quantification of the CNV lesion area.

For the quantification of laser-induced CNV, the eyes were enucleated and fixed in a 4% paraformaldehyde solution in phosphate-buffered saline. Choroidal flat mounts were generated and stained with Alexa Fluor^®^ 594-conjugated IB4 (1:100 dilution; Invitrogen) overnight at 4 °C^11^. Images of CNV lesions were obtained using a fluorescence microscope (Olympus); the exposure and gain were kept constant for all samples. In each whole-mount image, the number of pixels in the area showing CNV was measured using ImageJ software (NIH) as reported previously^10^.

### Cell culture

Human umbilical vein endothelial cells (HUVECs; Lonza, Walkersville, MD) were cultured from passages 3–6 in endothelial growth medium-2 (Lonza) or endothelial basal medium (EBM; Lonza) containing 1% fetal bovine serum (FBS; Lonza) at 37 °C in a humidified atmosphere of 95% air and 5% CO_2_.

### In vitro angiogenesis assay

To analyze tube formation, cells were seeded onto Matrigel (BD Bioscience, Bedford, MA)-coated 24-well plates and treated with EBM containing 1% FBS supplemented with or without 50 ng/mL VEGF (R&D Systems, Minneapolis, MN) and 1 μM evogliptin. After 6 h of incubation, the tube lengths in four random microscope fields were measured and used to quantify the tube lengths. In the scratch wound migration assay, confluent cell monolayers seeded in 24-well plates were scratched using pipette tips and washed with phosphate-buffered saline to remove dislodged cells. The cells were then incubated for 24 h with EBM containing 1% FBS and supplemented with or without VEGF (50 ng/mL) and evogliptin (1 μM). Cell migration was observed using an optical microscope and quantified by measuring the area covered by cells that had migrated from the wound edges. To analyze spheroid sprouting, spheroids were generated in EBM containing autoclaved methyl cellulose (Sigma-Aldrich) and grown in hanging drops for 24 h. Spheroids were then harvested and embedded in collagen gel in 24-well plates; the collagen gel was composed of collagen (R&D Systems), 10× DMEM (Sigma-Aldrich), 10% FBS, and sodium hydroxide (Sigma-Aldrich). After 1 h of gelation at 37 °C, EBM with or without supplemental VEGF (50 ng/mL) and evogliptin (1 μM) was added to the top of the collagen gels. Following 24 h of incubation, cell sprouting was observed using an optical microscope and quantified by measuring the sprout lengths of each spheroid. For experiments with a adenosine 5′-diphosphate ribosylation factor (Arf)-directed guanosine 5′-triphosphatase (GTPase)-activating protein (Arf-GAP) inhibitor, QS11 (1 μM; Abcam, Cambridge, UK) was added 24 h prior to VEGF stimulation.

### Western blot analysis

Cells and retinal tissues were lysed with lysis buffer supplemented with a proteinase inhibitor cocktail and phosphatase inhibitors, and the lysates were separated using sodium dodecyl sulfate-polyacrylamide gel electrophoresis (SDS-PAGE). Blots were hybridized with the appropriate primary IgG; antibodies against the following were used: phosphorylated VEGF receptor 2 (p-VEGFR2, Y1214: OriGene, Rockville, MD; Y1175: Cell Signaling Technology, Danvers, MA; Y1054: Millipore, Billerica, MA), phosphorylated Src (p-Src, Cell Signaling Technology), phosphorylated p38 mitogen-activated protein kinase (p-p38 MAPK, Cell Signaling Technology), phosphorylated focal adhesion kinase (p-FAK, Cell Signaling Technology), phosphorylated caveolin-1 (p-caveolin-1, BD Bioscience), VEGFR2 (Cell Signaling Technology), Src (Santa Cruz Biotechnology, Inc., Dallas, TX), p38 MAPK (Cell Signaling Technology), FAK (Cell Signaling Technology), caveolin-1 (R&D Systems), and β-actin (Sigma-Aldrich). This was followed by incubation with horseradish peroxidase-conjugated secondary immunoglobulin G (IgG). Immunoreactive bands were visualized using a chemiluminescent reagent (Amersham Biosciences, Piscataway, NJ). Densitometry was performed using ImageJ software (NIH).

### Immunocytochemistry

Cells growing on 2% gelatin-coated glass coverslips were treated for 24 h with EBM containing 1% FBS with or without VEGF (50 ng/mL) and evogliptin (1 μM). The cells were then fixed with 4% paraformaldehyde and permeabilized in 0.5% (w/v) Triton X-100. The cells were blocked with 10% normal serum and incubated overnight at 4 °C with primary IgGs against p-VEGFR2 (Millipore), p-caveolin-1 (R&D Systems), and p-FAK (Cell Signaling Technology), followed by incubation with fluorescent secondary IgGs. Nuclei were stained with 4ʹ,6-diamidino-2-phenylindole. Images were acquired using a confocal microscope (Carl Zeiss, Jena, Germany). All images shown are representative of at least three independent experiments.

### Arf6-guanosine 5ʹ-triphosphate (GTP) pulldown assay

Arf6-GTP pulldown assays were performed using an Arf6 activation assay kit (Cell Biolabs, Inc., San Diego, CA) according to the manufacturer’s instructions. Briefly, cells were treated with EBM with or without VEGF (50 ng/mL), evogliptin (1 μM), and QS11 (1 μM). After 1 h of incubation, cells were washed with ice-cold phosphate-buffered saline and lysed with ice-cold lysis buffer containing phosphatase inhibitors. The lysates were centrifuged, and the supernatants were incubated with GGA3-conjugated agarose beads at 4 °C for 1 h with gentle agitation. The beads were then washed and resuspended in reducing SDS-PAGE sample buffer. These bead solutions were then separated using SDS-PAGE, and the blots were hybridized with anti-Arf6 IgG, followed by horseradish peroxidase-conjugated secondary IgG. A fraction of the cell lysate was retained for the quantification of total Arf6 in that sample.

### Statistical analysis

GraphPad Prism software (GraphPad Software Inc., San Diego, CA) was used to analyze the data. Statistical significance was evaluated using an unpaired Student’s *t* test or one-way analysis of variance with Bonferroni’s post hoc multiple comparison test. The data are presented as the mean ± standard error of the mean (SEM); *p* values < 0.05 were used to indicate significance. The number of samples is indicated by *n*.

## Results

### Intravitreal administration of evogliptin alleviated pathological retinal NV in mice with OIR

**Fig. 1:**
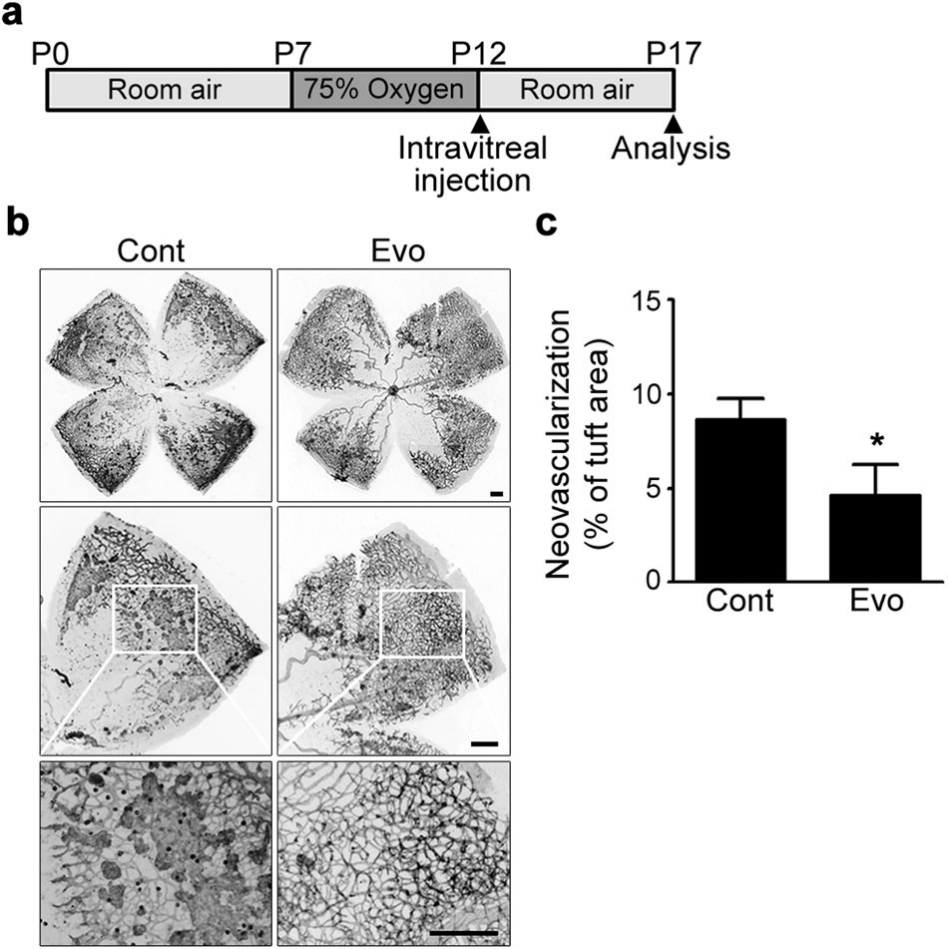
Intravitreal administration of evogliptin alleviated pathological retinal NV in mice with OIR. **a** Schematic illustration of OIR experiments. On P12, mice received a single intravitreal injection of evogliptin (Evo; 10 μg in 1 μL of DMSO) or DMSO (1 μL; contralateral control (Cont)). Five days later (on P17), the eyes were harvested for further analysis. **b** Representative images of whole-mounted retinas of mice with OIR. The retinal vasculature was visualized by staining with IB4 (black). High-magnification views of the boxed areas are shown at the bottom. Scale bars = 100 μm. **c** Retinal NV in mice with OIR was quantified as a percentage of the number of pixels in the neovascular tuft compared to the total number of pixels in the entire retina. Data are presented as the mean ± SEM (unpaired Student’s *t* test, *p < 0.05, *n* > 11 mice).

As the present study aimed to investigate the direct local effect of evogliptin, a DPP-4 inhibitor, on pathological NV in the eye, evogliptin was intravitreally injected into mice with OIR that displayed the hallmark features of aberrant retinal NV in PDR. On P17, the extent of pathological retinal NV was assessed by quantifying neovascular tufts in the retina (Fig. 1a). Compared to DMSO-injected control eyes, which displayed pronounced tuft formation in the retina, the eyes of evogliptin-injected mice exhibited a significant decrease in retinal NV (Fig. 1b, c). We also confirmed the antiangiogenic effect of evogliptin using a different murine model of pathological ocular NV in the choroid due to laser photocoagulation-induced rupture of Bruch’s membrane. Oral administration of evogliptin significantly reduced laser-induced CNV in mice compared to that in the control (Supplementary Fig. I). Moreover, the single intravitreal injection of evogliptin immediately after laser injury substantially alleviated pathological NV in the choroid (Supplementary Fig. II). These data indicate that evogliptin directly inhibited pathological ocular NV in murine models.

### Evogliptin suppressed VEGF-induced angiogenesis in endothelial cells

**Fig. 2:**
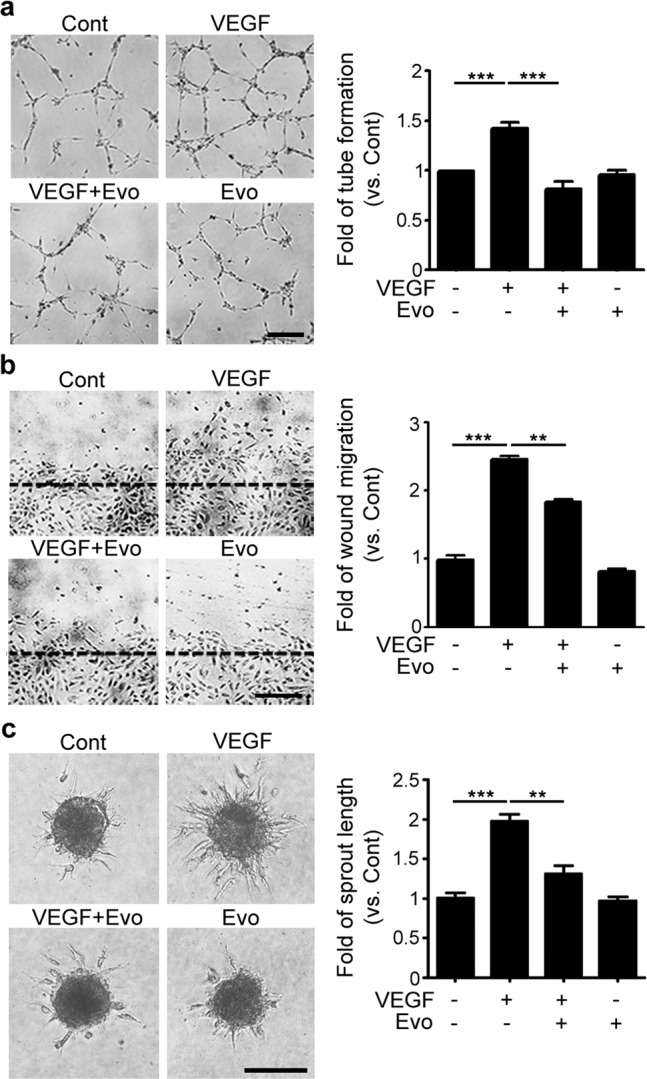
Evogliptin suppressed VEGF-induced angiogenesis in endothelial cells. Evogliptin (Evo) blocked VEGF-induced increases in the **a** tube formation, **b** scratch wound migration, and **c** spheroid sprouting of HUVECs. Cells were treated with or without Evo (1 μM) and VEGF (50 ng/mL). Tubular length, the relative area covered by migrating cells from the wound edges (black dashed lines), and the length of sprouts were measured and normalized to the values of the corresponding controls (Cont). Data are presented as the mean ± SEM (one-way ANOVA with Bonferroni post hoc multiple comparison test, **p < 0.01, ***p < 0.001; *n* > 4 for tube formation assay, *n* = 3 for scratch wound migration assay, and *n* > 20 for spheroid sprouting assay). Scale bars = 200 μm.

Because VEGF is a key angiogenic factor in pathological NV, we sought to test whether evogliptin would inhibit VEGF-induced angiogenesis in endothelial cells. In vitro angiogenesis experiments (tube formation, scratch wound migration, and spheroid sprouting assays) using HUVECs were performed in the presence or absence of VEGF and evogliptin. Figure 2 shows that pretreatment with evogliptin substantially blocked the VEGF-induced increase in endothelial cell migration, tube formation, and sprouting. However, evogliptin itself did not affect the basal angiogenic activity of HUVECs. These results suggest that evogliptin efficiently reduced VEGF-induced angiogenesis in endothelial cells.

### Evogliptin inhibited the VEGF-mediated angiogenic signaling pathway in endothelial cells

**Fig. 3:**
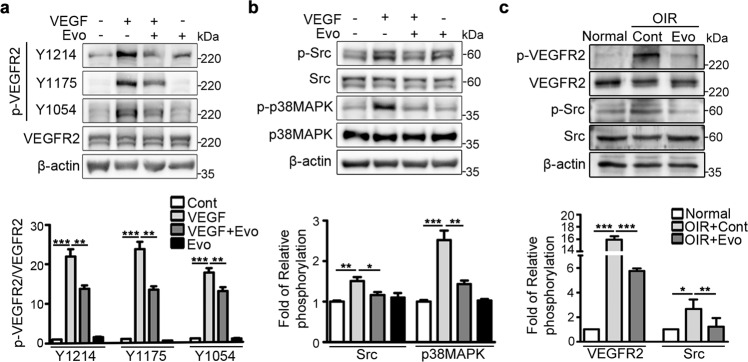
Evogliptin inhibited the VEGF-mediated angiogenic signaling pathway in endothelial cells. **a**, **b** Representative western blotting images and densitometric analysis of phosphorylated **a** VEGFR2 (p-VEGFR2) and **b** downstream signaling molecules (p-Src and p-p38 MAPK) in HUVECs. Cells were treated with or without evogliptin (Evo) and VEGF for 15 min, and cell lysates were subjected to western blotting. Band intensities of phosphorylated proteins were normalized to those of the total proteins (three independent experiments). **c** Representative western blotting images and densitometric analysis of p-VEGFR2 and p-Src levels in the retinal tissues of normal mice and mice with OIR. On P12, mice with OIR received a single intravitreal injection of Evo or DMSO (Cont). On P13, the retinal tissues of normal mice and mice with OIR were harvested for western blotting (*n* = 3 mice). All data are presented as the mean ± SEM (one-way ANOVA with Bonferroni post hoc multiple comparison test, *p < 0.05, **p < 0.01, ***p < 0.001).

We then examined whether evogliptin attenuates the VEGF-induced phosphorylation of VEGFR2 and its downstream signaling molecules. Western blot analysis revealed that VEGF induced the phosphorylation of VEGFR2 at multiple tyrosine sites (Y1214, Y1175, and Y1054) and that of Src and p38 MAPK, its downstream signaling molecules (Fig. 3a, b). However, treatment with evogliptin significantly reduced the VEGF-induced phosphorylation of VEGFR2, Src, and p38 MAPK. To ascertain whether a similar change in VEGF signaling would occur in vivo, we examined the phosphorylation levels of VEGFR2 and Src in the retinal tissues of normal mice and mice with OIR. Compared to normal mice, control mice with OIR treated with DMSO displayed a significant increase in p-VEGFR2 and p-Src levels in their retinal tissues (Fig. 3c). However, mice with OIR that received an intravitreal injection of evogliptin on P12 exhibited a substantial decrease in p-VEGFR2 and p-Src to levels, which were comparable to those in normal mice. These in vitro and in vivo data indicate that evogliptin significantly suppressed VEGF-mediated angiogenic signaling in endothelial cells.

### Evogliptin inhibited the VEGF-induced formation of focal adhesion (FA) and phosphorylation of FAK and caveolin-1 in endothelial cells

**Fig. 4:**
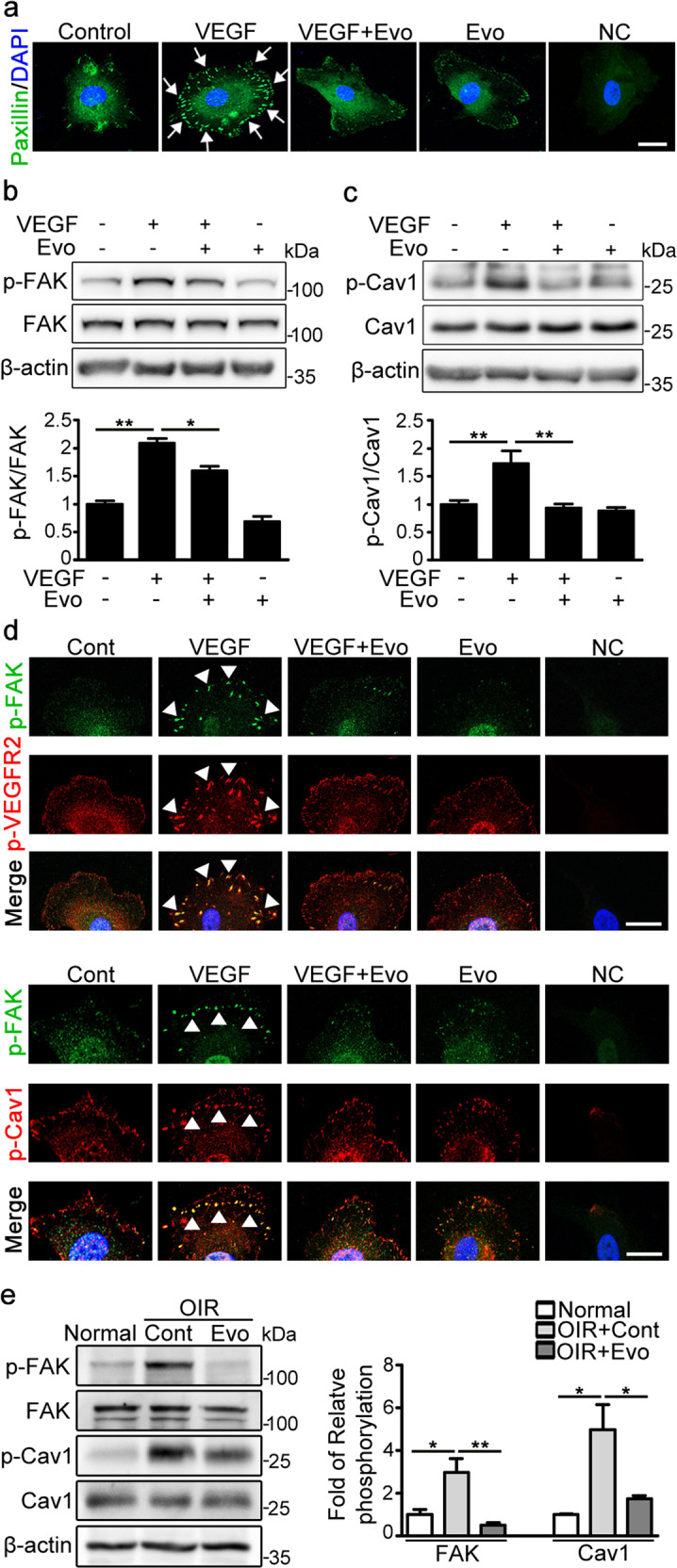
Evogliptin inhibited the VEGF-induced formation of focal adhesions and phosphorylation of FAK and caveolin-1 in endothelial cells. **a** Immunocytochemical detection of paxillin (green)-positive focal adhesions (white arrows) in HUVECs treated with or without evogliptin (Evo) and VEGF. **b**, **c** Representative western blotting images and densitometric analysis of phosphorylated **b** FAK (p-FAK) and **c** caveolin-1 (p-Cav1) in HUVECs treated with or without Evo and VEGF. Cell lysates were subjected to western blot analysis, and the signal intensity of the protein bands was determined by densitometry (three independent experiments). Band intensities of phosphorylated proteins were normalized to those of the total proteins. **d** Immunocytochemical detection of the colocalization of p-VEGFR2 and p-FAK and that of p-FAK and p-Cav1 in HUVECs treated with or without Evo and VEGF. The arrowheads indicate the spatial overlap between p-VEGFR2 (red) and p-FAK (green) and between p-FAK (green) and p-Cav1 (red). **e** Representative western blotting images and densitometric analysis of p-FAK and p-Cav1 in the retinal tissues of normal mice and mice with OIR. On P12, mice with OIR received a single intravitreal injection of Evo or DMSO (Cont). On P13, the retinal tissues of normal mice and mice with OIR were harvested for western blotting (*n* = 3 mice). All data are presented as the mean ± SEM (one-way ANOVA with Bonferroni post hoc multiple comparison test, *p < 0.05, **p < 0.01, ***p < 0.001). In **a** and **d**, the nuclei were stained with 4ʹ,6-diamidino-2-phenylindole (blue), and nonspecific IgGs were used as a negative control (NC). Representative immunocytochemical images were selected from three independent experiments with similar results. Scale bars = 25 μm.

Because cellular movement during migration, tube formation, and sprouting is mediated by cellular interaction with the extracellular matrix at FA sites, we examined the effect of evogliptin on FA formation in VEGF-treated endothelial cells. Immunocytochemical analysis revealed that VEGF-treated HUVECs formed abundant, paxillin-positive FAs near the cell periphery. However, cells treated with both evogliptin and VEGF formed far fewer paxillin-positive FAs than those treated with only VEGF (Fig. 4a). We then determined the phosphorylation levels of FAK, which is a key regulator of FA assembly for cellular movement and morphogenesis. Treatment with evogliptin substantially abrogated the VEGF-induced increase in FAK phosphorylation in endothelial cells (Fig. 4b). Activated Src phosphorylates caveolin-1 at Tyr14, which is required to stabilize the localization of FAK within FAs, thereby regulating cellular migration and invasion^12^. Given that evogliptin suppresses VEGF-induced Src phosphorylation, we investigated the effect of evogliptin on the phosphorylation of caveolin-1 and the subcellular localization of p-caveolin-1. Via western blotting, we found that the VEGF-induced phosphorylation of caveolin-1 at Tyr14 was substantially inhibited by treatment with evogliptin (Fig. 4c). Immunofluorescence results also revealed that VEGF strongly promoted the colocalization of p-VEGFR2 with p-FAK and that of p-caveolin-1 with p-FAK, which was significantly inhibited by cotreatment with evogliptin (Fig. 4d). In line with these in vitro data, western blot analysis of retinal tissues also revealed that the phosphorylation levels of both FAK and caveolin-1 were markedly reduced in the retinal tissues of mice with OIR treated with evogliptin compared to those of control mice with OIR (Fig. 4e).

### Evogliptin suppressed VEGF-induced angiogenesis by blocking Arf6 activation in endothelial cells

Rho small GTPase family members regulate cell adhesion, spreading, and migration. Several studies have shown that DPP-4 inhibitors reduce the migration of monocytes and splenic CD4-positive T cells by inactivating Rac1 small GTPase, a Rho family member^13–15^. In addition, it was recently reported that the small GTPase Arf6 is involved in the VEGF-induced activation of downstream signaling molecules and Rac small GTPase in endothelial cells^16,17^. These findings prompted us to explore the effect of evogliptin on Arf6 during VEGF-induced angiogenesis.

**Fig. 5:**
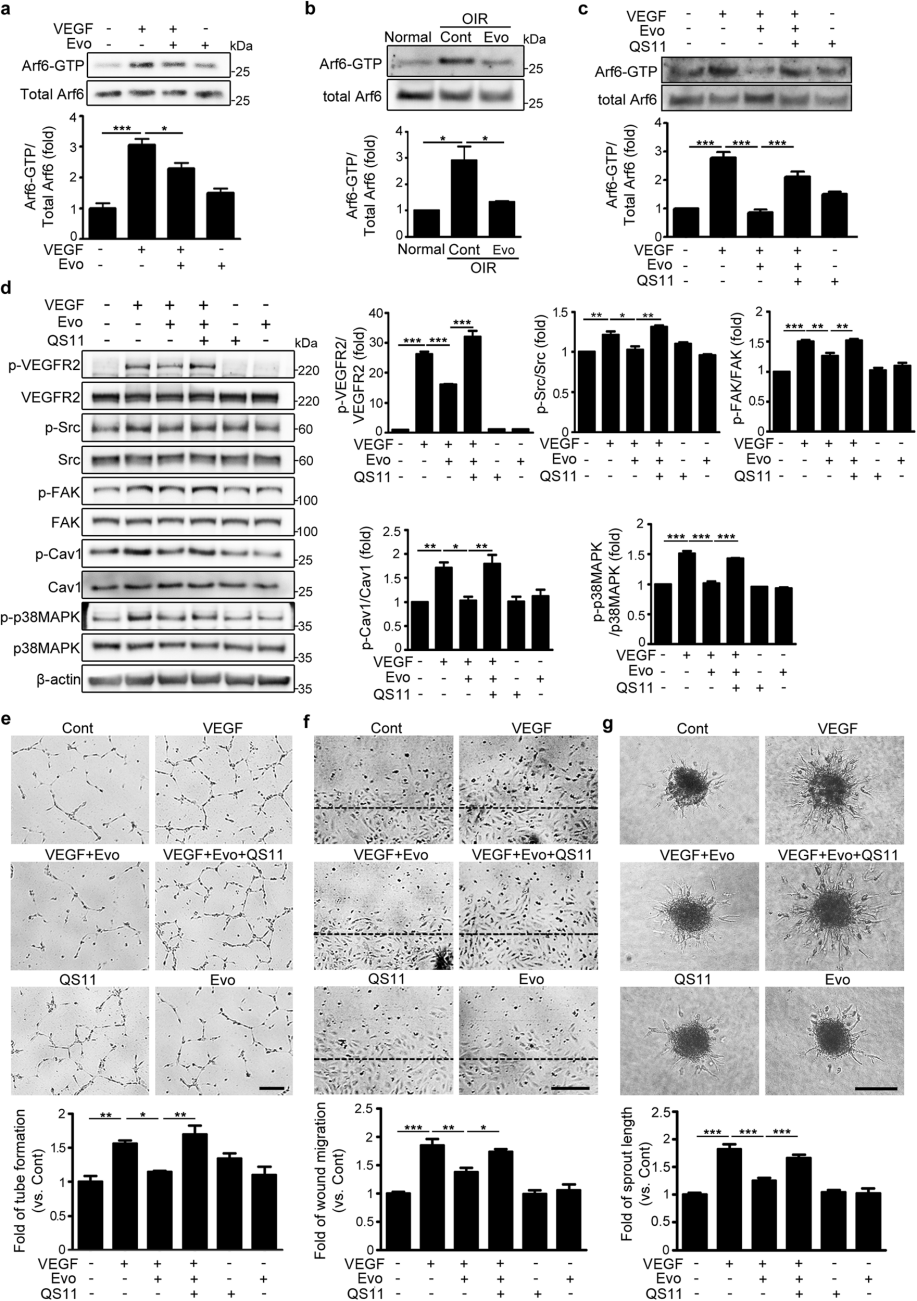
Evogliptin suppressed VEGF-induced angiogenesis by blocking Arf6 activation in endothelial cells. **a**, **b** Evogliptin (Evo) inhibited Arf6 activation in VEGF-treated HUVECs and mice with OIR. **a** Arf6-GTP levels were measured in HUVECs treated with or without Evo and VEGF for 15 min (three independent experiments). **b** Mice with OIR received a single intravitreal injection of Evo or DMSO (Cont) on P12, and retinal tissues were harvested from normal mice and mice with OIR on P13 (*n* = 3 mice). In **a** and **b**, cell and tissue lysates were prepared for Arf6-GTP pulldown analysis. The signal intensity of the protein bands was determined by densitometry, and the band intensities of phosphorylated proteins were normalized to those of the total proteins. **c**, **d** Treatment with an Arf-GAP inhibitor abolished the inhibitory effect of Evo on the VEGF-induced activation of **c** Arf6 and **d** the angiogenic signaling pathway in HUVECs. Cells pretreated with QS11 were incubated with or without Evo and VEGF for Arf6-GTP pulldown and western blotting (three independent experiments). **e**–**g** Treatment with an Arf-GAP inhibitor abolished the inhibitory effect of Evo on the VEGF-induced angiogenic activity of endothelial cells. **e** Tube formation, **f** scratch wound migration, and **g** spheroid sprouting assays were performed with HUVECs treated with or without QS11, Evo, and VEGF. Tubular length, the relative area covered by migrating cells from the wound edges (black dashed lines), and the length of sprouts were measured as previously described (*n* = 4 for tube formation assay, *n* = 8 for scratch wound migration assay, and *n* > 19 for spheroid sprouting assay). Scale bars = 200 μm. All data are presented as the mean ± SEM (one-way ANOVA with Bonferroni post hoc multiple comparison test, *p < 0.05, **p < 0.01, ***p < 0.001).

VEGF-treated endothelial cells showed significantly higher levels of the GTP-bound active form of Arf6 (Arf6-GTP) than the untreated control cells (Fig. 5a). However, treatment with evogliptin substantially abrogated the VEGF-induced increase in Arf6-GTP levels. Western blot analysis of the retinal tissues of normal mice and mice with OIR also revealed that a single intravitreal injection of evogliptin substantially decreased the Arf6-GTP levels in the retinal tissues of mice with OIR (Fig. 5b). To confirm that this inhibitory effect of evogliptin on VEGF-induced angiogenesis was principally due to the suppression of Arf6 activation, we sought to determine the effect of pretreatment with a small-molecule inhibitor of Arf-GAP, QS11, on the ability of evogliptin to inhibit VEGF-induced activation of downstream signaling molecules^17,18^. Pretreatment with QS11 increased Arf6-GTP levels in HUVECs treated with both VEGF and evogliptin to levels comparable to those in cells treated only with VEGF (Fig. 5c). Moreover, pretreatment with QS11 restored the VEGF-induced phosphorylation of VEGFR2, Src, caveolin-1, FAK, and p38 MAPK in evogliptin-treated HUVECs (Fig. 5d). Furthermore, pretreatment with QS11 blocked evogliptin-induced decreases in the tube formation, wound migration, and sprouting activities of VEGF-treated HUVECs (Fig. 5e–g). These data suggest that the inhibition of VEGF-mediated angiogenesis by evogliptin mainly occurs by the blockade of VEGF-induced Arf6 activation in endothelial cells.

## Discussion

In the present study, we have demonstrated that an intravitreal injection of the DPP-4 inhibitor evogliptin ameliorated pathological retinal NV in a murine model representing the vascular pathological features of PDR and reduced VEGF-mediated angiogenesis in human primary endothelial cells. Our in vitro and in vivo investigations of endothelial cells and retinal tissues, respectively, have verified that this antiangiogenic effect of evogliptin was mediated by inhibition of the VEGF-induced activation of Arf6, resulting in blockade of the activation of downstream signaling molecules, particularly those involved in cell adhesion and migration. These findings suggest that evogliptin exerts a direct inhibitory effect on VEGF-mediated pathological retinal NV and imply that evogliptin treatment could be beneficial to individuals with PDR, independent of its glucose-lowering effect.

Recently, several clinical data have revealed that DPP-4 activity might be associated with DR progression; hence, DPP-4 inhibitors may exert protective effects against DR that are independent of their glucose-lowering effect^4–6^. However, it is not known whether DPP-4 inhibitors can directly prevent or decrease pathological NV in the eye. Jung et al. assessed the efficacy of gemigliptin in preventing retinal NV in mice with OIR. They found that the oral administration of gemigliptin significantly reduced tuft formation in the retina and attributed the antiangiogenic effect of gemigliptin to a decrease in plasminogen activator inhibitor-1 expression^19^. Kolibabka et al. showed that systemic administration of linagliptin reduced pathological retinal NV in OIR in both C57BL/6J and *Glp1r*-knockout mice, indicating that the antiangiogenic effect of linagliptin in mice with OIR is independent of the glucagon-like peptide-1 receptor. The authors also revealed that linagliptin treatment inhibited the phosphorylation of extracellular signal-regulated kinases^20^. These findings demonstrate that DPP-4 inhibitors have potent antiangiogenic activities and directly interfere with pathological NV in the retina; however, the mechanism underlying this antiangiogenic effect of DPP-4 inhibitors has not been clearly elucidated.

In the present study, we found that evogliptin exerted an antiangiogenic effect by inhibiting VEGF-induced activation of the small GTPase Arf6. Evogliptin significantly suppressed Arf6 activation in VEGF-treated human endothelial cells and the retinal tissues of mice with OIR. Moreover, the restoration of Arf6 activity with a chemical inhibitor of Arf-GAP completely abrogated the inhibitory effect of evogliptin on the VEGF-induced activation of downstream signaling molecules and increase in endothelial angiogenic activities. This demonstrates that the inhibition of VEGF-induced angiogenesis by evogliptin is principally due to the suppression of Arf6 activation. Arf6 is a key regulator of angiogenesis induced by VEGF and other growth factors^16,21,22^. Zhu et al. reported that Arf6 activation was required for maximal VEGF/VEGFR2 signaling activation in human vascular endothelial cells and that the inhibition of Arf6 activation completely blocked the VEGF-induced phosphorylation of VEGFR2 and its downstream signaling molecules^16^. In mice with OIR, blockade of Arf6 activation using SecinH3, an inhibitor of an Arf GTP exchange factor (GEF), substantially inhibited pathological retinal NV^17^. Moreover, Lin et al.^23^ have shown that Arf6 plays a pivotal role in both developmental and pathological lymphangiogenesis, particularly in the VEGF-C-mediated migration and sprouting of lymphatic endothelial cells. These reports support our finding that the inhibition of VEGF-induced angiogenesis by evogliptin occurred through the blockade of Arf6 activation. However, it is unclear how evogliptin regulates Arf6 activation. DPP-4 interacts with integrin β1 to induce the phosphorylation of integrin β1 at S785, which is necessary for integrin β1 to act as an adhesion molecule^24,25^. Indeed, linagliptin, a DPP-4 inhibitor, blocked the phosphorylation of integrin β1 in the kidneys of diabetic mice, thereby preventing profibrotic endothelial–mesenchymal transition^26^. Phosphorylated integrins regulate cellular adhesion and migration by controlling the association with GEF and/or GAP of Arf^27^. Considering the role of DPP-4 and Arf in the integrin-mediated cellular adhesion process, it is plausible that evogliptin might regulate Arf6 activity by modulating integrin phosphorylation and, consequently, the recruitment of Arf-GAP or Arf-GEF. This is the subject of ongoing investigation in our laboratory.

Our results also demonstrate that evogliptin suppressed the phosphorylation of subcellular signaling molecules involved in cell adhesion and migration. In human primary endothelial cells, evogliptin treatment substantially inhibited VEGF-induced cell migration, sprouting, and morphogenesis into a capillary tubular structure, all of which require FA assembly for initial cellular interaction with the extracellular matrix. Indeed, evogliptin treatment attenuated VEGF-induced FA formation and the activation of FA assembly-related subcellular signaling molecules, including FAK and caveolin-1, and their association at FA sites in endothelial cells. Similarly, evogliptin significantly reduced the phosphorylation of FAK and caveolin-1 in the retina of mice with OIR. VEGF-induced phosphorylation of caveolin-1 is mediated by Arf6 activation, which also supports our finding that evogliptin inhibits Arf6 activation by VEGF^22^.

To summarize, the current study provides novel mechanistic evidence of the antiangiogenic activity of the potent DPP-4 inhibitor evogliptin. The results of our study indicate that evogliptin directly interfered with pathological retinal NV by blocking VEGF-induced Arf6 activation in endothelial cells. The present findings suggest that evogliptin may provide additional therapeutic benefits in diabetic patients with PDR beyond glycemic control.

## Supplementary information

Supplementary Figures
