# Supplementary material for: Evogliptin, a dipeptidyl peptidase-4 inhibitor, attenuates pathological retinal angiogenesis by suppressing vascular endothelial growth factor-induced Arf6 activation
Source: Exp Mol Med. 2020 Oct 14;52(10):1744–53. doi: 10.1038/s12276-020-00512-8 (PMC8080693; doi:10.1038/s12276-020-00512-8)
Supplement: Supplementary file 1 — Supplementary Figures [file 12276_2020_512_MOESM1_ESM.doc]

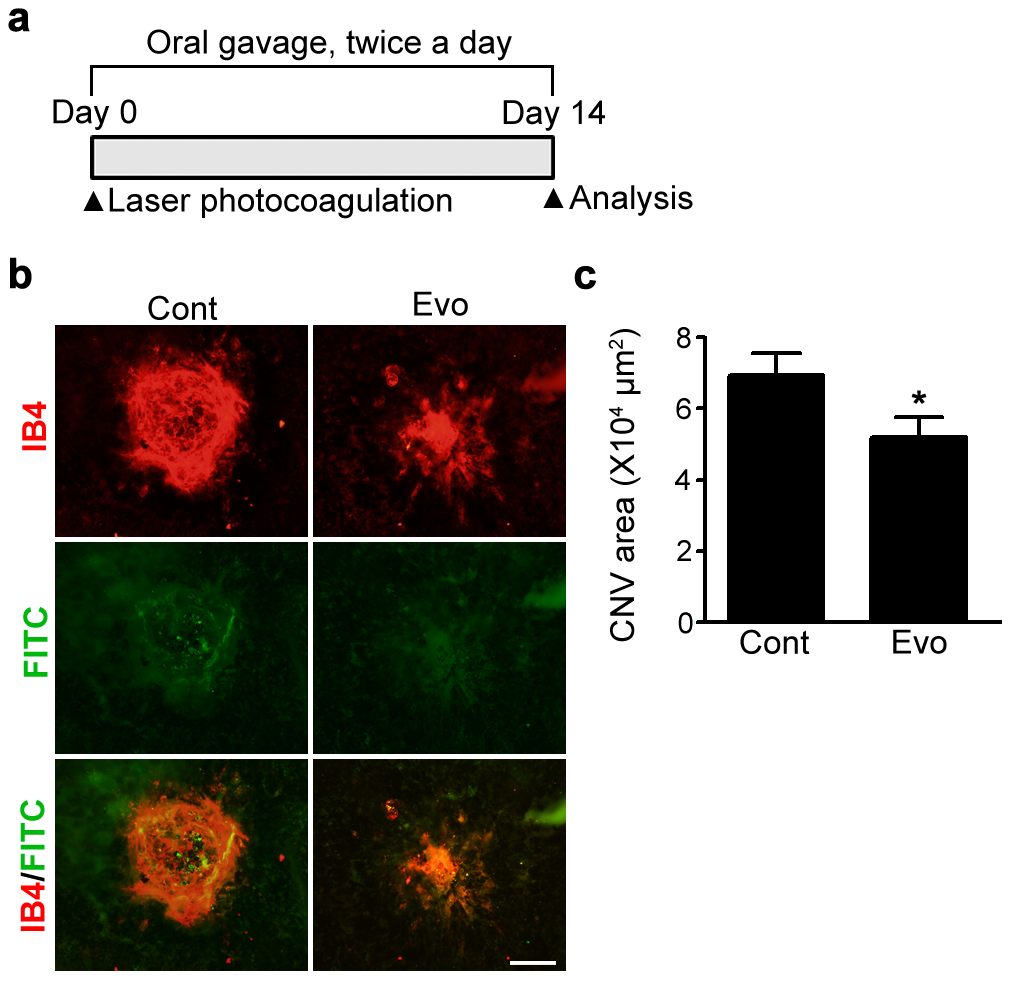


**Supplementary Figure I. Oral administration of evogliptin reduced pathological CNV in mice**.

(a) Schematic diagram of laser-induced CNV experiments. Immediately after laser photocoagulation, the mice received oral administration of evogliptin (Evo; 20 mg/Kg/day in 0.5% methylcellulose) or vehicle control (Cont; 0.5% methylcellulose) in twice a day. Two weeks later, the eyes were harvested for further analysis. (b) Representative images of flat-mounted choroids with CNV lesions. On day 14 after laser photocoagulation, the mice were perfused with fluorescein isothiocyanate (FITC)-dextran (green) and choroidal flat mounts were stained with IB4 (red). Scale bar = 100 μm. (c) The areas of CNV lesions in (b) were quantified by measuring the fluorescence intensity of images with IB4-positive areas. Data are presented as the mean ± SEM (unpaired Student’s *t*-test, **p* < 0.05, *n* > 23 laser spots).


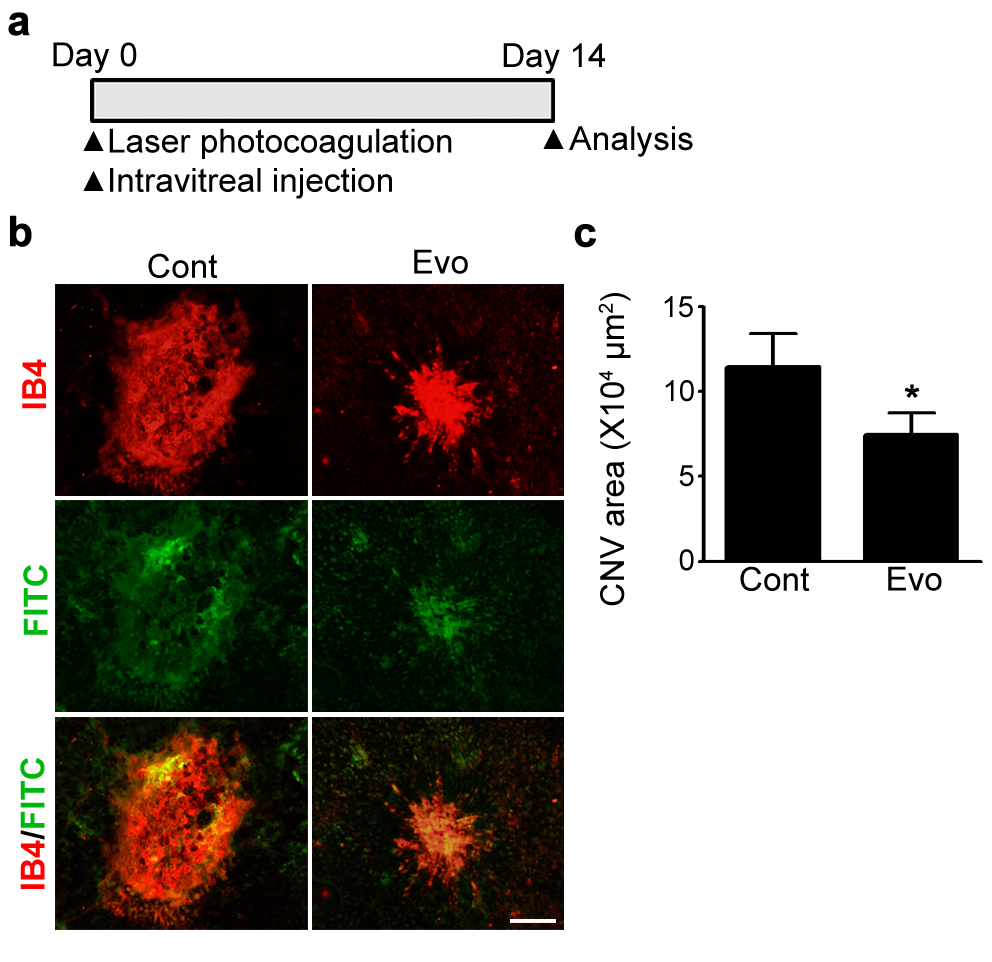


**Supplementary Figure II. Intravitreal administration of evogliptin alleviated pathological CNV in mice.**

(a) Schematic diagram of laser-induced CNV experiments. Immediately after laser photocoagulation, the mice received a single intravitreal injection of evogliptin (Evo; 10 μg in 1 μL of DMSO) or DMSO (1 μL; contralateral control (Cont)). Two weeks later, the eyes were harvested for further analysis. (b) Representative images of flat-mounted choroids with CNV lesions. On day 14 after laser photocoagulation, mice were perfused with FITC-dextran (green) and choroidal flat mounts were stained with IB4 (red). Scale bars = 100 μm. (c) The areas of CNV lesions in (b) were quantified by measuring the fluorescence intensity of images with IB4-positive areas. Data are presented as the mean ± SEM (unpaired Student’s *t*-test, **p* < 0.05, *n* > 18 laser spots).
